# Deficits in Inhibitory Control in Smokers During a Go/NoGo Task: An Investigation Using Event-Related Brain Potentials

**DOI:** 10.1371/journal.pone.0018898

**Published:** 2011-04-22

**Authors:** Maartje Luijten, Marianne Littel, Ingmar H. A. Franken

**Affiliations:** Institute of Psychology, Erasmus University Rotterdam, Rotterdam, The Netherlands; University of Granada, Spain

## Abstract

**Introduction:**

The role of inhibitory control in addictive behaviors is highlighted in several models of addictive behaviors. Although reduced inhibitory control has been observed in addictive behaviors, it is inconclusive whether this is evident in smokers. Furthermore, it has been proposed that drug abuse individuals with poor response inhibition may experience greater difficulties not consuming substances in the presence of drug cues. The major aim of the current study was to provide electrophysiological evidence for reduced inhibitory control in smokers and to investigate whether this is more pronounced during smoking cue exposure.

**Methods:**

Participants (19 smokers and 20 non-smoking controls) performed a smoking Go/NoGo task. Behavioral accuracy and amplitudes of the N2 and P3 event-related potential (ERP), both reflecting aspects of response inhibition, were the main variables of interest.

**Results:**

Reduced NoGo N2 amplitudes in smokers relative to controls were accompanied by decreased task performance, whereas no differences between groups were found in P3 amplitudes. This was found to represent a general lack of inhibition in smokers, and not dependent on the presence of smoking cues.

**Conclusions:**

The current results suggest that smokers have difficulties with response inhibition, which is an important finding that eventually can be implemented in smoking cessation programs. More research is needed to clarify the exact role of cue exposure on response inhibition.

## Introduction

Several contemporary models of addiction highlight the role of impulsivity and executive functioning in the development and maintenance of addiction [1]–[8]. A core component of executive functioning is response inhibition which is generally defined as the ability to adaptively suppress behavior when environmental contingences demand this [9]. It has been proposed that poor response inhibition in substance-dependent individuals is associated with difficulties to resist the consumption of a substance especially when exposed to highly salient substance-related cues [1].

Reduced response inhibition has been observed in several substances dependent patient populations including alcohol [10], cocaine [11], and opioid [12] dependent patients. Some studies have also investigated response inhibition in smokers. In these studies, inhibitory control was generally assessed by means of behavioral paradigms, such as Go/NoGo tasks. In the Go/NoGo task, participants have to respond as quickly as possible to frequently occurring ‘Go’ stimuli, and to inhibit responses to infrequent ‘NoGo’ stimuli. Results of studies on response inhibition in smokers have been inconsistent. That is, some studies have found response inhibition during a Go/NoGo task to be impaired in smokers relative to controls [13] whereas other studies did not find this group difference in performance on the Go/NoGo task [14], nor on other behavioral tasks measuring response inhibition [15], [16]. The recording of electroencephalographic (EEG) activity during response inhibition has been suggested to yield more sensitive indices (i.e., event-related potentials, ERPs) of response inhibition and may therefore clarify the inconsistent results. Two major ERP components have been reported to be enhanced for NoGo trials as compared to Go trials suggesting that these reflect changes in brain activity related to response inhibition in a Go/NoGo task. The first of these ERP-components is the NoGo N2 which is a negative wave that emerges approximately 200–300 ms after stimulus presentation and has maximum peaks on frontal scalp sites. Mounting evidence suggests that the NoGo N2 amplitude is a valuable measure for response inhibition. The NoGo N2 amplitude has been consistently found to be related to behavioral outcomes of inhibitory control on Go/NoGo tasks [17] irrespective of the stimulus modality used in these tasks [18], [19]. Although, Go and NoGo trials differ with respect to the overt motor response, which could influence the difference between Go and NoGo N2 amplitudes, it has been found that theNoGo N2 is not restricted to tasks requiring these overt motor responses [20], furthermore a modulation of the N2 ERP by response inhibition requirements has been observed in other inhibition-related paradigms besides the Go/NoGo task [21]–[23].

The second ERP component that has been associated with response inhibition research, is the NoGo P3 which is a positive wave that emerges circa 300–500 ms after stimulus onset and has a more central distribution. There are some concerns about the exact role or meaning of P3 amplitudes in response inhibition processes [17], [24]. In contrast to the NoGo N2, the NoGo P3 does not seem to be consistently related to response inhibition on a behavioral level. However, some studies show a clear relationship between NoGo P3 amplitude and behavioral outcomes of response inhibition tasks [20], [25]. Moreover, because the P3 is a rather late ERP-component (>300 ms) it has been suggested that it does not reflect the initial reflexive stage of the inhibition process but rather a later stage of the inhibition process that is closely related to the actual inhibition of the motor system in the premotor cortex [21], [26]. In any event, both decreased NoGo P3 and N2 amplitudes have been reported in various populations with reduced inhibitory control such as children with ADHD [27], [28] and impulsive violent offenders [29] suggesting that both ERP components are adequate indices of inhibitory processes in impulsive populations.

Few studies have used ERPs to investigate response inhibition with ERPs in substance-dependent patients [30]–[34] and, to our knowledge, only one of these studies has focused on smokers [33]. Remarkably, with the exception of the study by Yang et al. [32], analyses of all these studies were confined to the P3 amplitude whereas studies in other psychiatric populations have usually investigated both the N2 and P3 amplitudes [27]–[29], [35]–[40]. ERP studies investigating response inhibition in substance-dependent patients have generally found NoGo P3 amplitudes to be reduced [31], [33], [34] as compared to healthy controls. However, in heroin patients only the NoGo N2 amplitude appeared to be reduced; no differences were found on the NoGo P3 [32].

All the above-mentioned studies investigated general response inhibition in addicted individuals by using affectively neutral task paradigms. It has been proposed, however, that the reactivity to conditioned drug-related stimuli and processes of executive functioning may impact each other in a synergistic way [1], [1,7]. This means that persons with a stronger reactivity towards drug-related cues may experience more problems with inhibiting their behavior. Over the course of addiction drug-related stimuli become extremely attractive to the addicted person and tend to grab the attention [3], [41]. A recent study demonstrated that participants' attentional bias for alcohol-related words was positively correlated with reduced inhibitory control in decision-making, particularly when the decisions were related to obtaining alcohol [42]. Altogether, a reciprocal relation between the attention grabbing properties of drug cues and inhibitory control has been proposed suggesting that decreased inhibitory control may be more enhanced during direct exposure to drug-related stimuli [3].

To test the idea that inhibitory control in substance-dependent individuals is particularly impaired in the presence of substance-related cues, the current study investigated response inhibition to both neutral and smoking-related cues in smokers and non-smoking controls. For this purpose a novel Go/NoGo paradigm was developed including smoking and neutral pictures. It is expected that smokers will generally show reduced response inhibition as compared to non-smoking controls. More specifically, on a behavioral level, it is expected that smokers will make more mistakes when they have to inhibit their response to infrequent NoGo stimuli. On an electrophysiological level we expect that N2 and P3 amplitudes during NoGo trials will be decreased in smokers as compared to non-smokers. Finally, we expect these effects to be more pronounced on trials which include smoking-related stimuli.

## Methods

### Participants

Nineteen smokers (mean age = 21.36 years, *SD* = 1.98, 14 male) and 20 non-smoking controls (mean age = 21.55 years, *SD* = 2.18, 14 male) participated in this study. Exclusion criteria were (a) the current abuse of a substance (other than nicotine for the smoking group), and (b) the current presence of a physical or psychiatric illness. There were no significant differences between the groups in mean age, *t*(37) = .27; ns, or gender ratio, χ^2^ (1, *n* = 39) = .07; ns. Smokers smoked at least 10 cigarettes per day (*M* = 17.95, *SD* = 5.88; range 10–30) for a duration of at least two years (*M* = 5.74, *SD* = 3.53, range = 2–17). Fagerström scores (FTND) were suggestive of medium levels of nicotine dependence, *M* = 5.05, *SD* = 2.27, range = 0–8 [43], [44]. Non-smokers had smoked ten or less cigarettes in their lifetime (*M* = 1.22, *SD* = 2.34, range = 0–10). Participants were undergraduate students, who received course credits or a financial compensation for their participation. The study was conducted in accordance with the Declaration of Helsinki and all procedures were carried out with the adequate understanding and written informed consent of the subjects. The study protocol was approved by the Ethics Committee of the Institute of Psychology of the Erasmus University Rotterdam.

### Instruments

Breath carbon monoxide concentration was measured using a Micro+ Smokerlyzer (Bedfort Scientific Ltd., Rochester, UK) in order to objectively identify smokers and non-smokers. Next to the FTND, smokers also completed the brief version of the Questionnaire of Smoking Urges [45] to assess their subjective craving for a cigarette.

### Task paradigm

**Figure 1:**
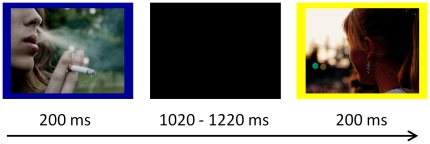
Example of a Go and NoGo trial combined with smoking neutral pictures in the smoking cue Go/NoGo task.

A smoking-related Go/NoGo task was developed for the aim of the current study. In this task participants were presented with a series of pictorial stimuli with a smoking or non-smoking-related content. Each picture was displayed for 200 ms and had a blue or yellow frame (see figure 1 for an example of a smoking and non-smoking trial). The frame color indicated whether a stimulus was a Go or a NoGo trial. The attribution of the frame color to Go versus NoGo trials was counterbalanced across participants. Each stimulus was followed by a black screen for a randomly varying duration between 1020 ms and 1220 ms. Participants were instructed to respond to the pictures in Go trials by pressing a button with the right index finger as fast as possible, and to withhold their response in the NoGo trials. They were explicitly instructed to maintain accuracy during the whole task. The task consisted of 112 different smoking-related pictures and 112 non-smoking-related pictures. Smoking-related pictures displayed smoking related objects (e.g., lighter, ashtray etc) or scenes of people engaged in smoking behavior, whereas non-smoking-related pictures displayed neutral items or scenes of people engaged in non-smoking behavior. Each picture was presented for four times during the whole task, once as a NoGo stimulus and three times as a Go stimulus. This means that 25% of all trials were NoGo trials and that the proportion of smoking and non-smoking pictures in the task was equal (i.e., 112 NoGo trials per picture category and 336 Go trials per picture category). The order of picture content (smoking versus neutral) was completely randomized and the order of trial type (Go versus NoGo) was quasi randomized such that at most four Go and two NoGo trials were presented consecutively. Before the start of the actual task, participants were given to opportunity to practice in 23 practice trials, involving additional non-smoking pictures. At four time moments during the task, participants were given the opportunity to take a short break. Total task duration was about 22 minutes, depending on the length of the breaks.

### Procedure

Smokers were instructed to abstain from smoking for at least one hour before the start of the experiment. This short period of smoking deprivation was introduced in order to reduce the acute effects of nicotine on ERP amplitudes [46], [47] without introducing withdrawal effects. Participants approved participation by signing informed consent. The CO breath sample was taken and questionnaires were completed. Subsequently, participants were seated in a comfortable EEG chair in a light and sound-attenuated room. Electrodes were attached and task instructions were explained. Participants performed the smoking Go/NoGo tasks during EEG recording. Smokers completed the QSU-brief a second time at the end of the experiment.

### EEG recording and data reduction

The EEG was recorded using the Biosemi Active-Two amplifier system (Biosemi, Amsterdam, the Netherlands) from 34 scalp sites (positioned following the 10–20 International System with two additional electrodes at FCz and CPz) with active Ag/AgCl electrodes mounted in an elastic cap. Six additional electrodes were attached to the left and right mastoids, to the two outer canthi of both eyes (HEOG), and to an infraorbital and a supraorbital region of the right eye (VEOG). All signals were digitalized with a sample rate of 512 Hz and 24-bit A/D conversion with a bandpass of 0–134 Hz. Data were off-line re-referenced to computed mastoids. Off-line, EEG and EOG activity was filtered with a bandpass of 0.10–30 Hz (phase shift-free Butterworth filters; 24 dB/octave slope). Data were segmented in epochs of 1 second (200 ms before and 800 ms after response or stimulus presentations). After ocular correction [48] epochs including an EEG signal exceeding ±75 µV were excluded from the average. The mean 200 ms pre-response or pre-stimulus period served as baseline. After baseline correction, average ERP waves were calculated for artifact-free trials at each scalp site for correct and incorrect responses separately. Segments with incorrect responses (miss for GO trials or false alarm for NoGo trials) were excluded from EEG analyses. The N2 was defined as the most negative value within the 200–300 ms time interval after stimulus onset and was studied at a cluster of frontocentral electrodes, including Fz, FC1, FC2, FCz and Cz [49]. The P3 was defined as the most positive value within the 300–500 ms time interval after stimulus onset. The P3 was studied at a cluster of central electrodes, including FCz, Cz, C3, C4 and CPz [49]. The mean number of analyzable Go and NoGo epochs for smoking pictures was 248.50 and 56.00 respectively and 250.57 and 55.26 for non-smoking pictures. One non-smoker was excluded from ERP analyses because of less than 10 artifact free ERP epochs in at least one of the task conditions. This participant was included in all remaining data analyses.

### Statistical analysis

The difference in self-reported craving before and after task performance was analyzed by means of a paired samples *t*-test. Repeated Measures Analyses of Variance (RM-ANOVA; with Greenhouse-Geisser adjusted *p*-values) were applied to analyze behavioral outcomes of performance on the Go/NoGo task, as well as ERP indices of response inhibition. The between-subjects factor in all RM-ANOVA's was Group (smokers versus non-smokers). Two-level within-subject factors were of interest, namely (a) Inhibition (Go versus NoGo), and (b) Picture (smoking versus non-smoking pictures). A Group×Inhibition×Picture RM-ANOVA was employed to analyze the behavioral accuracy during the Go/NoGo task, and a Group×Picture RM-ANOVA was chosen to analyze reaction times in Go trials. Electrode (Fz, FC1, FC2, FCz, Cz for N2 and FCz, Cz, C3, C4, and CPz for P3) was included as a five-level within subject factor in the ERP analyses. That is, a Group×Inhibition×Picture×Electrode RM-ANOVA was performed for the ERP analyses. Post-hoc tests for interactions were performed only for interactions including the between subject factor Group. A Bonferroni correction for multiple comparisons was applied in all post-hoc analyses. Finally,Spearman correlation coefficients were calculated for the number of cigarettes per day on the one hand and NoGo accuracy rates and average cluster peaks across all electrodes for the NoGo N2 and P3 on the other hand.

## Results

### Breath CO levels and questionnaires

In line with expectancies, smokers had a higher breath concentration of carbon monoxide (CO; in parts per million, *M* = 12.89, *SD* = 7.15) as compared to non-smoking controls (*M* = 1.15, *SD* = 1.04), *t*(37) = 7.27, *p*<0.001. Subjective craving in smokers increased significantly from the start (*M* = 37.53, *SD* = 10.02) to the end (*M* = 46.05, *SD* = 9.79) of the experiment, *t*(18) = 4.35, *p*<.001.

### Behavioral data

**Figure 2:**
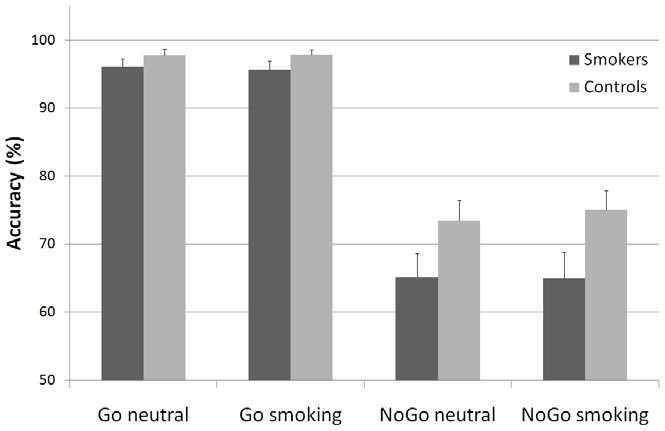
Accuracy rates in smokers and non-smoking controls on the smoking cue Go/NoGo task.

The accuracy rates for both the smoking and non-smoking group on the smoking-related Go/NoGo task are displayed in Figure 2.A robust main effect of Inhibition was found, *F*(1,37) = 184.55, *p*<0.001 showing that participants were less accurate on NoGo trials (69.63% versus 96.86% respectively). There was also a main effect for Group, *F*(1,37) = 4.12, *p* = .05, which indicated that overall task performance was less accurate in smokers than in non-smoking controls (80.47% 86.03%, respectively). A trend to significance was found for the Group×Inhibition interaction, *F*(1,37) = 3.27, *p* = 0.08. Post-hoc *t*-tests revealed that, particularly on NoGo trials, smokers performed less accurate than non-smoking controls (*p* = 0.05; 65.05% versus. 74.23%), whereas there was no difference on accuracy between the groups for Go trials. No main or interaction effects of Picture were found for accuracy of responding. We additionally performed two seperate RM-ANOVA's for Go and NoGo accuracy scores because of differences in the distibution for Go and NoGo accuracy which may lead to subsequent differences in the magnitude of effects for Go and NoGo accuracy. Results showed the same pattern as the combined analysis. A main effect for Group was found for NoGo accuracy. *F*(1,37) = 4.02, *p* = 0.05 confirming that smokers were less accurate than controls on NoGo trials. No difference on accuracy between groups was found for Go trials. No main or interaction effects of Picture were found for accuracy of responding in either NoGo or Go trials.

With regard to the reaction time data, a main effect of Picture was found, *F*(1,37) = 6.28, *p*<.05 indicating that participants generally responded faster to smoking-related Go trials than to neutral Go trials (*M* = 259.69 ms versus*M* = 261.89 ms). No other significant effects were found for reaction times. No significant correlations were found between the number of cigarettes smoked per day and accuracy rates for NoGo trials.

### N2 amplitudes

**Figure 3:**
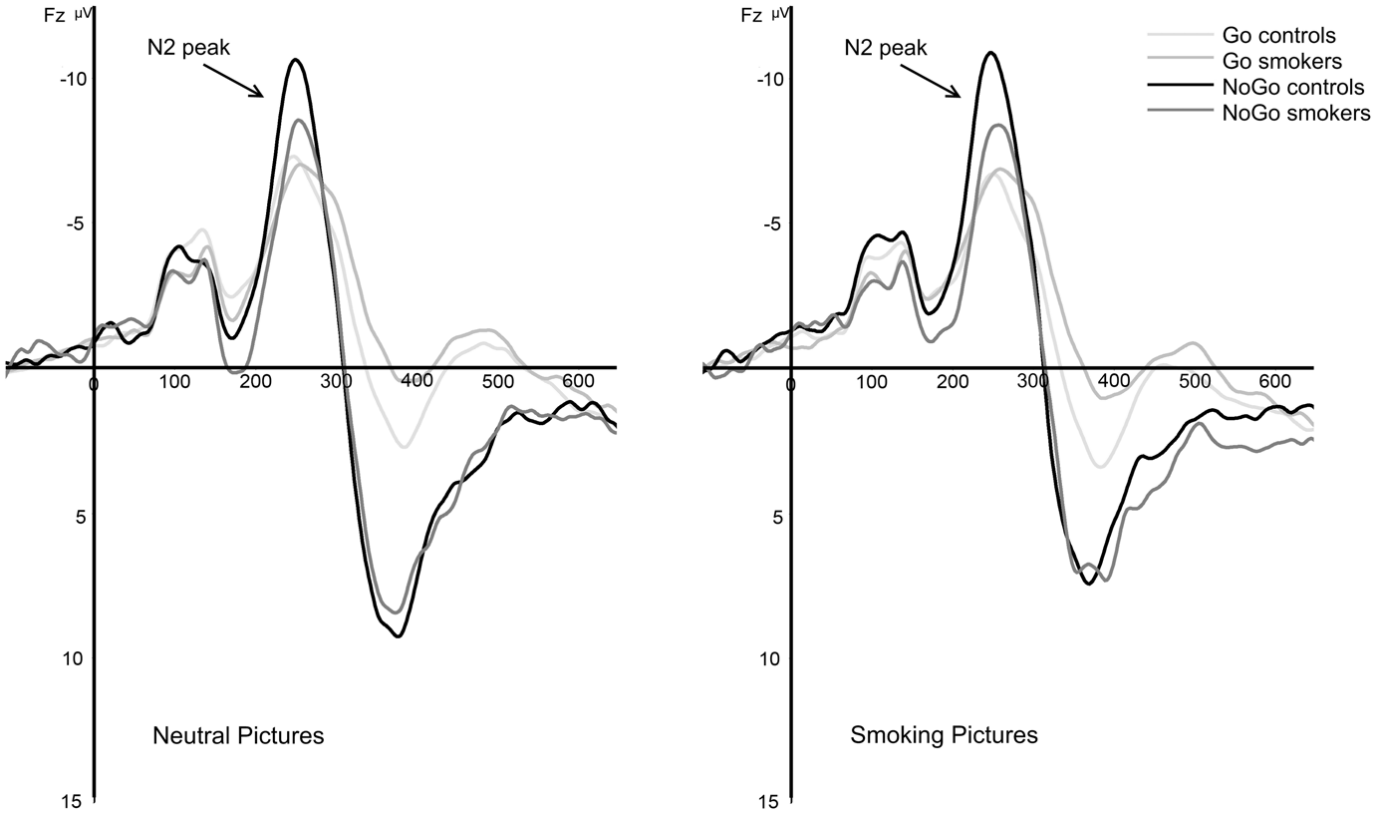
Grand-average stimulus-locked waveforms for neutral and smoking pictures at Fz for correct Go and NoGo trials in smokers and non-smoking controls.

The N2 amplitude for smoking-related and neutral pictures in both groups is displayed in Figure 3. In line with the hypotheses, a robust main effect was found for Inhibition, *F*(1,36) = 36.83, *p*<.001 on the N2 component at the frontocentral electrode cluster. This result demonstrates that N2 amplitudes were generally larger for NoGo trials than for Go trials. Importantly, a Group×Inhibition interaction effect was found, *F*(1,36) = 6.31, *p* = .017. Post-hoc *t*-tests indicated that only on NoGo trials the N2 was significantly reduced in smokers as compared to non-smoking controls (*p* = .046), whereas there were no between-group differences on N2 amplitude in response to Go trials. Furthermore, a main effect for electrode was found, *F*(4,144) = 25.67, *p*<.001. No Picture-related main or interaction effects were found for the N2 component. No significant correlations were found between the number of cigarettes smoked per day and the cluster combined N2 peak amplitudes for NoGo trials.

### P3 amplitudes

**Figure 4:**
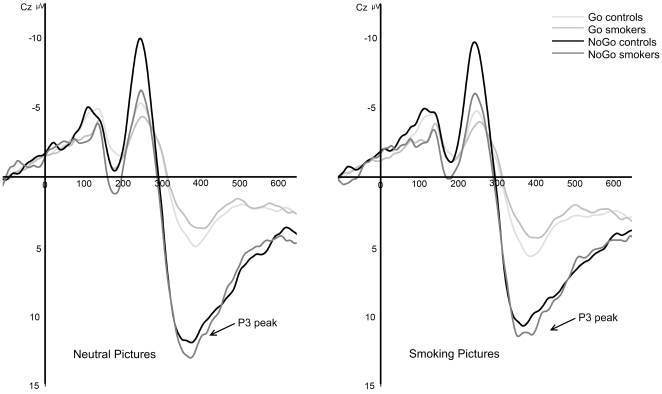
Grand-average stimulus-locked waveforms for neutral and smoking pictures at Cz for correct Go and NoGo trials in smokers and non-smoking controls.

Figure 4 shows the P3 amplitudes for smoking and neutral pictures in both groups. As expected for the P3 at the central electrode cluster, a robust main effect was found for Inhibition, *F*(1,36) = 138.85, *p*<.001. This result indicates that the P3 peaks were generally larger for NoGo trials than for Go trials. Furthermore, a main effect for electrode was found, *F*(4,144) = 18.73, *p*<.001. No other significant main or interaction effects including Group were found for P3 amplitude. No significant correlations were found between the number of cigarettes smoked per day and the cluster combined P3 peak amplitudes for NoGo trials.

## Discussion

The main purpose of the present study was to investigate differences in response inhibition on a behavioral as well as on an electrophysiological level using a smoking-modified Go/NoGo paradigm in combination with the recording of event-related potentials. Consistent with the notion that the N2 reflects an inhibitory process, the N2 was significantly enhanced on NoGo trials as compared to Go trials. More importantly and in line with our primary hypothesis differences between smokers and non-smokers were found on both behavioral and electrophysiological indices of response inhibition. That is, performance on the Go/NoGo task was generally less accurate in smokers than non-smokers in such that smokers displayed significantly more difficulties to inhibit their responses in NoGo trials. This deficit in general response inhibition was also reflected in reduced N2 amplitudes in NoGo trials in smokers as compared to non-smokers.

Previous studies on inhibitory control in smokers, which used behavioral paradigms, such as the Go/NoGo task generally yielded inconsistent results [13]–[16]. The difficulty level of the task of the current study, however, was different than previous studies. The Go/NoGo task that was used in the present study placed high demands on inhibition capacities because stimulus presentation was fast and NoGo trials were infrequent. This is supported by the fact that 31.4% of the NoGo trials resulted in commission errors in the current study while this is usually much lower (e.g., 5% percent in Dinn et al. [14]). In addition, the present study was the very first to include the N2 component as an additional index of inhibitory control in smokers. The NoGo N2 amplitude is an index of response inhibition which is believed to be more sensitive than behavioral outcomes in the Go/NoGo paradigm [50], [51]. The fact that reduced inhibitory control was found in behavioral accuracy as well on the N2 component of the ERP provides support for the hypothesis that there is a general shortcoming in response inhibition in smokers relative to non-smokers. It must be noted, however, that the current study design does not allow drawing conclusions on causality. It may be that reduced inhibitory control is the result of prolonged nicotine dependence, for example via abnormalities in the dopamine system, or that reduced inhibitory control is a predisposition to start smoking. The latter interpretation may be more convincing according to the results of the current study because of the lack of association between measures of inhibitory control and nicotine exposure (i.e. the number of cigarettes smoked per day). This association could be expected if reduced inhibitory control is the result of a modulation of brain systems by nicotine intake.

With regard to P3, enlarged amplitudes in NoGo trials than Go trials were observed confirming that, like the N2 amplitude, P3 amplitude is related to response inhibition processes. However, contrary to N2 and behavioral accuracy, no differences between groups were found in NoGo P3 amplitude. It has been suggested that, whereas the NoGo N2 might be related to an early stage of the response inhibition process, the NoGo P3 might reflect a later stage of the inhibition process that is closely related to the actual inhibition of the motor system [26]. Accordingly, the present study results suggest that the reduced inhibitory control in smokers reflect a dysfunctional activation of inhibitory processes at an early stage of cortical processing while later stages of the inhibition process may be intact. This is in line with findings in heroin dependent patients [32] but in contrast to previous findings in alcohol dependent patients [31], ecstasy polydrug users [34] and smokers [33]. Unfortunately, the latter studies did not investigate N2 amplitudes making complete comparisons with the present study impossible. Furthermore, the diverse characteristics of the Go/NoGo paradigms used in these studies might have contributed to differential findings [31]. For example, the probability of NoGo trials, and thereby the demand on response inhibition capacities, varies largely among these ERP studies just as in the studies investigating behavioral accuracy. Furthermore, it is not clear whether previous studies separated successful and unsuccessful trials in examining P3 amplitudes which is important because P3 amplitudes are influenced by inhibition success or failure [26].

The present study was the first that investigated not only general response inhibition in smokers, but specific response inhibition towards smoking-related stimuli as well. Several authors suggest that inhibitory control in substance-dependent individuals is especially reduced when exposed to drug-related cues and that this eventually may contribute to compulsive cue-elicited drug intake [3], [7], [52]. The findings in the current study could not confirm that reduced inhibitory control in smokers is more pronounced in the presence of smoking cues. In fact, the current findings show that inhibitory control is reduced in smokers during smoking cue exposure and during neutral affective conditions suggestingthat these deficits found in smokers are of a more general category, which may be in favor of the diagnostic value and theoretical importance of the Go NoGo task paradigm. Furthermore, these results imply that decreased inhibitory control may not only influence smoking related behavior but also other impulsive and possibly maladaptive behaviors. This idea is supported by the high proportion of smokers in, for example, conduct disorder [53] and problem gambling [6].However, the influence of drug cue-exposure on levels of inhibitory control should be further investigated in future studies. Possibly, the overall reduced inhibitory capacity of smokers, which was observed in the present study, reflects a general effect of nicotine craving. That is, controlling the craving elicited by the smoking-related pictures during the task might have required cognitive resources [54] which might have resulted in an overall reduced inhibitory capacity. In the present study, smokers reported significantly increased craving after task performance as compared to before showing that smokers had to deal with increasing levels of craving evoked by the smoking-related pictures used in the Go/NoGo task. One way to examine if craving has a general effect on performance of the smoking-modified Go/NoGo paradigm, could be to present the stimuli to participants in a blocked design, with one block of neutral pictures being presented first, followed by a block of smoking-related pictures to measure response inhibition under low and high conditions of craving separately.

In conclusion, results of the current study showed reduced inhibitory control in smokers both at behavioral and physiological measures. Decreased N2 amplitudes for NoGo trials were accompanied by reduced accuracy for NoGo trials. However, the hypothesis that reduced response inhibition would be more pronounced for smoking related cues could not be confirmed. These results suggest that smokers have difficulties with inhibitory control, which might be an important factor in the initiation and continuation of smoking behaviors as well as relapse in smoking behaviors. These findings can eventually be implemented in smoking cessation programs.
